# Promoting Awareness of Key Resources for Evidence-Informed Decision-making in Public Health: An Evaluation of a Webinar Series about Knowledge Translation Methods and Tools

**DOI:** 10.3389/fpubh.2016.00072

**Published:** 2016-04-22

**Authors:** Jennifer Yost, Jeannie Mackintosh, Kristin Read, Maureen Dobbins

**Affiliations:** ^1^Faculty of Health Sciences, School of Nursing, McMaster University, Hamilton, ON, Canada; ^2^National Collaborating Centre for Methods and Tools, McMaster University, Hamilton, ON, Canada

**Keywords:** public health, evidence-informed decision-making, knowledge translation, webinar, evaluation

## Abstract

The National Collaborating Centre for Methods and Tools (NCCMT) has developed several resources to support evidence-informed decision-making – the process of distilling and disseminating best available evidence from research, context, and experience – and knowledge translation, applying best evidence in practice. One such resource, the Registry of Methods and Tools, is a free online database of 195 methods and tools to support knowledge translation. Building on the identification of webinars as a strategy to improve the dissemination of information, NCCMT launched the Spotlight on Knowledge Translation Methods and Tools webinar series in 2012 to promote awareness and use of the Registry. To inform continued implementation of this webinar series, NCCMT conducted an evaluation of the series’ potential to improve awareness and use of the methods/tools within the Registry, as well as identify areas for improvement and “what worked.” For this evaluation, the following data were analyzed: electronic follow-up surveys administered immediately following each webinar; an additional electronic survey administered 6 months after two webinars; and Google Analytics for each webinar. As of November 2015, there have been 22 webinars conducted, reaching 2048 people in multiple sectors across Canada and around the world. Evaluation results indicate that the webinars increase awareness about the Registry and stimulate use of the methods/tools. Although webinar attendees were significantly less likely to have used the methods/tools 6 months after webinars, this may be attributed to the lack of an identified opportunity in their work to use the method/tool. Despite technological challenges and requests for further examples of how the methods/tools have been used, there is overwhelming positive feedback that the format, presenters, content, and interaction across webinars “worked.” This evaluation supports that webinars are a valuable strategy for increasing awareness and stimulating use of resources for evidence-informed decision-making and knowledge translation in public health practice.

## Introduction

The National Collaborating Centre for Methods and Tools (NCCMT) was established in 2007, through funding from the Public Health Agency of Canada, to support public health organizations and professionals across Canada to use the best available evidence in practice, program, and policy decisions (1). Since then, NCCMT has developed a suite of resources to promote the necessary knowledge and skills for evidence-informed decision-making (EIDM) – informing public health decisions through consideration of knowledge from the best available research evidence along with knowledge about community health issues and the local context, existing public health resources and the community and political climate (2). The aim in developing this suite of resources was to address the multitude of individual and organizational barriers to EIDM, such as: limited access to research evidence, knowledge and skills to access, interpret, evaluate, and synthesize research evidence (3–8), time (3–5, 8, 9), and financial resources (5, 7, 9).

One of the resources created by NCCMT is the Registry of Methods and Tools or *Registry*. The Registry is an online, searchable, and freely accessible collection of methods and tools to support knowledge translation – or, in other words, the process of synthesizing, disseminating, exchanging, and applying various types of knowledge (e.g., research evidence, knowledge of the local context) to decisions concerning health (10). The Registry is currently comprised of 195 methods and tools. *Methods* (*n* = 101) are processes, regular or systematic approaches, or sets of organized steps and *tools* (*n* = 94) are standardized products (e.g., instruments, surveys, and checklists). The methods and tools available in the Registry were identified through systematic searches of the published literature conducted during 2008–2009 and 2011. Moving forward, methods and tools for inclusion in the Registry have been identified through suggestions provided by NCCMT users and NCCMT team members.

All methods and tools in the Registry are assessed for relevance to knowledge translation and the Canadian public health context. Once deemed relevant, a summary is written for each method or tool using a standardized template for relevant areas (e.g., measurement, development, and availability) and then made available within the Registry. The Registry can be searched by registered and non-registered NCCMT users using an open text box or according to steps in the EIDM process (i.e., define, search, appraise, synthesize, adapt, implement, and evaluate) (11) and/or knowledge translation-related activities (e.g., organizational change, partnership evaluation, and priority setting). Further details about the Registry’s history and methodology, as well as how to use the Registry, are available at NCCMT’s website^1^ and in a previous publication by Peirson and colleagues (12).

A formative evaluation of the Registry was previously conducted in 2012 (12). Results of an online survey and individual interviews with NCCMT registered users provided areas for improving users experience with the Registry and indicated that the Registry is a valuable resource for EIDM by providing access to supports, which help to integrate knowledge from research evidence into public health decision-making (12). In addition, the link between several strategies to promote the Registry and an increase in the number of users accessing the Registry was also identified; such strategies included e-mail blasts, newsletter announcements, presentations at conferences, and webinars.

As NCCMT continues to use webinars to promote the Registry, it is important to comprehensively evaluate the potential of webinars to improve awareness and use of the methods and tools within the Registry.

## Background

With technology and its associated applications increasing the availability of information (13), webinars have been identified as a strategy to improve the sharing of information in a valuable way. A webinar, or Web-based seminar, is a presentation, lecture, workshop, or seminar transmitted over the Internet and includes the following characteristics: advanced registration, a presentation that includes voice with a presenter who is not visible, limited interaction with participants that typically follows the presentation, and archival of content (14). With such characteristics, webinars differ from webcasts, which are designed to push a pre-recorded (asynchronous) presentation to an audience, as well as interactive web conference meetings, which provide for synchronous sharing desktops and applications and communication between participants using the Internet (14).

Within the literature, there is general consensus about the positive aspects of webinars. For example, webinars improve the reach of information across time and geographic boundaries (14–22). This in turn can increase the number of people to whom information is shared, spanning different audiences and settings (15). Similarly, webinars may decrease the cost of disseminating information (13, 23), especially in terms of travel time when people are participating from different geographic locations (13). Along with consistent reports of overall positive experiences with webinars (14, 16, 19, 24, 25), the literature also suggests that webinars can stimulate awareness of and action in relation to information shared (23, 26).

Although the capability of webinars to promote interactivity between those producing and using information has been identified (18, 21), arguments that webinars limit interaction also exist (13, 14, 24). This is often noted in comparisons of webinars to face-to-face strategies (13, 14). Challenges with technology are another undesirable aspect of webinars that is consistently identified within the literature (13, 18). For example, attendees often experience challenges downloading webinar applications on their computer, as well as poor audio and Internet connectivity (13, 18). This being said, there is evidence that less technical support is required the more people engage in webinars (13). Furthermore, the results of an evaluation conducted by Buxton and colleagues (24) suggest that the timing and selection of topics that are of interest to attendees are more important than technology/Internet requirements in continued webinar participation.

Taking into consideration the existing literature describing webinars as an effective strategy for sharing information, as well as acknowledging the challenges associated webinars, NCCMT began a webinar series – *Spotlight on Knowledge Translation Methods and Tools* – in January 2012, to promote the methods and tools in the Registry. As such, the purpose of this paper is to describe a descriptive evaluation to identify (a) if the webinar series increases awareness and use of the methods and tools available in the Registry, as well as (b) areas for improvement and (c) “what worked” in implementing webinars.

## Methods

### Webinar Format

The webinars in the Spotlight series use common format components, including advance registration, 90 min synchronous presentation, use of polling questions, and dissemination of the PowerPoint presentation and audio recording following the webinar (see Table 1). Each Spotlight webinar features one method or tool from the Registry and is held on a day in the middle of the week (Tuesdays, Wednesdays, and Thursdays) at a consistent time (1:00 p.m. to 2:30 p.m. EST). The common agenda for each webinar is as follows: first a NCCMT Team Member begins the webinar with housekeeping items, overview of NCCMT, and introduction of the presenters (10 min). Then, the developer provides an overview of the method/tool (30 min), for example, why it was developed, how it is used, and how it can be applied in a public health context. Then, if available, a user story is shared that describes how the tool has been used within a public health department or regional health authority (20 min). The final 30 min of the webinar is open for webinar participants’ questions.

**Table 1:** **Spotlight on knowledge translation methods and tools webinar format components**.

• Advance registration
• Designated host and presenter(s)
• Voice presentation only
• 90 min synchronous presentation
○ Overview of NCCMT and presenter(s)
○ Overview of the method/tool
○ User story
○ Question and answer session
• Polling questions
• Archival of PowerPoint presentation and audio recording

To identify which method/tool will be featured in webinars the following approaches have been used: methods/tools that are highly accessed through the Registry (identified through Google Analytics), methods/tools for which a *user story* is available, and methods/tools addressing the different steps in the EIDM process (11). User stories are instances where we have become aware that a public health department or regional health authority in Canada has used a particular method/tool; either through NCCMT’s work with the health department or regional health authority or public health professionals contacting us to tell us that they have used a particular method/tool. Once the method/tool has been identified, we then contact the developer of the method/tool to determine their interest in presenting an overview of the method/tool during a webinar session and do our best to find a related user story.

### Webinar Promotion

To promote the webinar sessions, multiple methods are used. First, we create what we refer to as a *one-pager* about the webinar. This is a brief document, which includes the title of the webinar, the date/time of the webinar, the presenters names and pictures, two brief paragraphs identifying the method/tool being highlighted (providing a short summary of the method/tool and its applicability to public health), a link to the summary of the method/tool on our website, and the link to register for the webinar. This one-pager is then posted into the registration page for the webinar and informs promotion of the webinar via NCCMT’s Event Calendar,^2^ newsletter, and promotional Tweets. NCCMT’s weekly newsletter – the *Round Up* – is disseminated via e-mail weekly and is available in an online archive.^3^

### Webinar Resources

Two resources, the webinar platform and staff, are required for implementation of the webinars. First, with various platforms available to conduct webinars, we are currently using a platform that supports many aspects which we have found to be important in our experiences conducting webinars. Our current platform allows for participant registration and designation of a webinar host and presenter(s) separate from webinar attendees. The platform also facilitates sharing of PowerPoint slides, broadcasting of audio, recording of audio, use of polling questions, posting of messages by the host/presenter(s), posting of messages/questions by the webinar attendees, and collection of quantitative data related to polling questions. In our webinars, host/presenter(s) post messages to the attendees via the *chat* function. Messages typically include information about how to troubleshoot technology issues and the availability of the PowerPoint and audio following the webinar, as well as links to different information shared within the webinar. Using the chat and/or Q&A function, attendees can choose to post questions or messages and can choose whether their comments are seen by just the host/presenter(s) or by all attendees. Although our current platform does offer an audio option, we use separate teleconferencing services to deliver the audio to the webinar attendees. Only the host and the presenter(s) connect via teleconference; the audio from the teleconferencing service is then broadcast through the webinar platform to webinar attendees who have joined on their computer or other similar device.

In addition to the webinar platform, multiple NCCMT team members are involved in the implementation of the Spotlight webinar series. Prior to the webinar, a communications coordinator is responsible for promotional activities described above, including editing of the one-pager and developing Tweets (~2 h/webinar). A research assistant then provides all assistance related to the technological aspects of the webinar platform described above. This includes creating the webinar registration page and “day-of” webinar-related activities. Day-of related tasks include initiating the webinar platform, uploading of PowerPoint slides, programming and releasing polling questions, bridging the audio, posting relevant information in the chat (e.g., links to content mentioned in the presentation), responding to technical questions, and downloading all in-event data (~2.5 h/webinar). Overseeing and coordinating all activities related to the webinar is performed by a senior level team member (~3.5 h/webinar of staff time). Related to the preparation for the webinar, this team member establishes contact with method/tool developers presenting by e-mail and phone and works with the presenter to develop the one-pager and PowerPoint slides. This senior level team member also facilitates the webinar on the day-of, which involves welcoming attendees, introducing the presenter, introducing polling questions, and moderating the Q&A session at the end of the webinar. Following the webinar, they are then responsible for collating data collected.

### Further Dissemination Activities

Since developing these webinars, we have engaged various strategies to disseminate the information presented to public health professionals unable to attend the webinars, wanting to revisit a webinar they attended, or looking for more information about a particular method/tool. Initially, to disseminate this information and to accommodate the francophone members of our target audience, we posted translated PowerPoint slides and transcribed audio recordings in French. Then, to increase the accessibility and uptake of the information, we instead, developed *webinar companions*, which were brief, plain language summaries that distilled the key points of each webinar into a few pages. In evaluating the use of webinar companions, we found that the number of times the webinar companions were viewed was quite low. Therefore, since June 2015, we are translating and posting complete PowerPoint presentations in both English and French to our Slideshare account, as well as posting the full webinar presentation (including the audio recording) in English only to our YouTube account.

### Webinar Evaluation

Evaluating the Spotlight webinar series involves an analysis of various types of data collected from webinar attendees during and following the webinars to identify who the webinar attendees are, as well as awareness of and intention to use the method/tool featured in the webinar.

During the webinar, we use *polling questions* to gather information about the geographic location attendees are joining from and the current sector in which they work. Additional polling questions are then used at the discretion of the presenter(s) to ask webinar specific questions of the attendees. For example, one of the most common questions presenters choose to ask is about attendees’ familiarity with the method/tool. At the conclusion of each webinar, attendees are asked about their intended *next steps* following the webinar (i.e., access the method/tool, consider using the method/tool, tell a colleague about the method/tool). Although our current webinar platform allows us to quantitatively record responses to polling questions, this option was unavailable in the webinar platform that we previously used. As such, the demographic information related to the sector and Canadian provinces/territory in which webinar attendees work is qualitatively summarized.

In addition to answering polling questions during the webinar, webinar attendees are asked to complete a follow-up survey. Currently, the link to this online survey is shared with attendees at the end of the webinar and then e-mailed to webinar registrants immediately following the webinar; the survey remains open for 1 week with no reminders sent. The survey consists of 16 questions using a combination of nominal, Likert scale, and open-ended questions. These questions collect data on demographics, attendance at previous webinars, familiarity with the method/tool (among attendees and/or their organizations), attendees intended *next steps* following the webinar, and suggestions for improvement. The quantitative and qualitative data are summarized below, in aggregate, across all webinars.

In addition to this follow-up survey, from November 2012, to May 2013, we evaluated self-reported intention to use and actual use of the method/tools 6 months after two webinars (October 2012, *Promoting Action on Equity Issues: A Knowledge-to-Action Framework* and November 2012, *KT Planning Template*). Considered program evaluation, this evaluation was exempt from Hamilton Integrated Research Ethics Board (HIREB) review. Webinar registrants of these two webinars were sent an electronic survey 6 months after the webinars, with a 2-week reminder e-mail. This survey used a combination of seven nominal, Likert-scaled, and open-ended questions which asked respondents: if they used the method/tool presented in the webinar in their work and if so, how and how useful they found the method/tool. A Pearson Chi-square test was conducted on aggregate data to determine difference in percentage of those who reported that they intended to use the method/tool immediately following the webinar (data from follow-up survey) to the percentage of those who reported that they actually used the method or tool in the past 6 months (data from 6-month survey). The responses to the open-ended questions were also explored, coded, and grouped into themes. Themes identified during qualitative data analysis were compared with quantitative results to explain findings about use of the methods/tools.

Finally, we use Google Analytics to track the number of visits to the NCCMT webpage in the Registry for the method/tools featured in each webinar. Data are summarized below in aggregate for all webinars.

## Results

Since January 2012, we have conducted 22 webinars on 19 different methods/tools as part of the Spotlight series; 7 in 2012, 4 in 2013, 5 in 2014, and 6 as of November 2015. Among the 22 webinars, 2 featured two methods and 17 were focused on tools, reflecting all steps in the EIDM process and the majority of knowledge translation and related activities. Across all webinars, of the 3714 people who registered for the webinars 2048 people attended the webinars (55.1% attendance rate). Of these, *N* = 546 completed the follow-up survey immediately following the webinar.

### Demographics

Our webinars have reached people working in multiple sectors (public health, health in general, education, research, and at provincial/territorial and government/ministry levels) throughout all Canadian provinces and territories, as well as those working internationally. Results from the follow-up survey indicate that the majority of webinar attendees are public health nurses/registered nurses (12.4%), academic researchers (10.4%), and health promoters (9.9%) (*n* = 434) (see Table 2 for follow-up survey results).

**Table 2:** **Spotlight on knowledge translation methods and tools webinar follow-up survey results**.

Current role/position	*n* (%)		
Academic/researcher	46 (10.6)		
Administration	10 (2.35)		
Dietitian/nutritionist/RD	14 (3.2)		
Communications	7 (1.6)		
Consultant	20 (4.6)		
Data analyst	4 (0.9)		
Dental hygienist	4 (0.9)		
Epidemiologist/statistician	15 (3.5)		
Health inspector	1 (0.2)		
Health promoter	43 (9.9)		
Health planner/evaluator/analyst	22 (5.1)		
Information specialist/librarian	11 (2.5)		
KB/translation specialist	25 (5.8)		
Physician	11 (2.5)		
Policy analyst	29 (6.7)		
Program coordinator/planner	22 (5.1)		
Program evaluator	9 (2.1)		
Program manager/director	26 (6.0)		
Public health nurse/RN	54 (12.4)		
Student	6 (1.4)		
Supervisor	4 (0.9)		
Other	54 (11.7)		

	**Yes [*n* (%)]**	**No [*n* (%)]**	**Unsure [*n* (%)]**

Previously heard of the method/tool			
Respondent	177 (34.7)	315 (61.9)	17 (3.3)

	**Strongly agree/agree [*n* (%)]**	**Disagree/strongly disagree [*n* (%)]**	**Neither agree nor disagree [*n* (%)]**

Intend to visit the registry			
Respondent	362 (85.8)	11 (2.6)	49 (11.6)
Gained new insights about using the method/tool			
Respondent	163 (63.7)	73 (28.5)	20 (7.8)
Organization	101 (77.7)	7 (5.4)	22 (16.9)
Respondent and organization	226 (91.5)	9 (3.6)	12 (4.9%)
Previously used the method/tool			
Respondent	15 (8.2)	156 (85.7)	11 (6.0)
Organization	4 (4.7)	47 (55.3)	34 (40)
Respondent and organization	36 (16.4)	141 (64.1)	43 (19.5)
Intend to use the method/tool			
Respondent and organization	354 (71.5)	15 (3.0)	126 (25.5)

### Awareness of the Registry

The Spotlight webinar series increases awareness of the Registry. Results from the follow-up survey indicate that, 85.8% (*n* = 362) of respondents intend to visit the Registry to find resources for using research evidence in their work. This represents an increase from the 49.3% (*n* = 225) of respondents who indicated that they had not accessed the Registry prior to attending the webinar.

### Awareness and Use of the Registry Methods/Tools

Although the majority of respondents to the follow-up survey had not previously heard about the method/tool featured in the webinar (61.9%, *n* = 315), data from the follow-up survey indicate that both awareness and intention to use the method/tool is high. The majority indicated that through the webinar they gained insight into how the method/tool would be useful to their work (63.7%, *n* = 163), the work of their organization (77.7%, *n* = 101), and their work/work of their organization (91.5%, *n* = 226). In addition, 80.6% (*n* = 150) would share information about the method/tool with a colleague following the webinar.

Similar to the results of awareness, the self-reported current use of the method/tool by respondents (8.2%, *n* = 15), by their organization (4.7%, *n* = 4), or by respondents/respondents organizations (16.4%, *n* = 34) was also quite low on the follow-up survey; although in this survey 71.5% (*n* = 354) of respondents indicated that they intended to use the method/tool in their work following the webinar. However, the 6-month survey demonstrated respondents were significantly less likely to have actually used the method/tool in their work than they had intended. Of 305 webinar attendees, 27.5% completed the follow-up survey and 15.1% completed the 6 month survey. It was indicated by 92.6% of follow-up survey respondents (*n* = 50) that they intended to use the method/tool in their work, whereas only 37% (*n* = 17) reported actually using the method/tool within the 6 months since the webinar, in their work (*p* < 0.0001). The qualitative data from the 6-month survey help us to understand this result. We were able to determine that participants had not used the method/tool, because there had not been an identified opportunity in their work to use the method/tool. Additionally, Google Analytics data suggest that the webinars stimulate action about the featured method/tools with total page views across the methods/tools increasing from 66,965 in the 180 days before the webinar to 71,744 views in the 180 days following the webinar.

### Areas for Improvement

The follow-up survey also asks respondents to identify changes they would like to see in order to improve the webinar. Across all webinars, 169 suggestions for improvement were related to two primary categories – *user stories* and technology. Despite the majority of webinars providing a user story, respondents want more examples of how the methods/tools have been used. They provided suggestions such as additional Canadian examples and examples from different settings. Challenges with technology were also identified, with respondents experiencing minor to major technological issues. These are primarily difficulty in hearing the audio and being “blocked” from the webinar platform. Respondents identified the option of testing their connection in advance of the webinar as a way of overcoming these technological challenges.

### Positive Feedback

In addition to providing areas for improvement, the follow-up survey also asks about what respondents *liked most* about the webinar and 302 positive comments were identified. Strengths of the webinars can be synthesized according to four categories: (1) format, (2) the presenters, (3) content, and (4) interaction. First, participants appreciate the clear, simple, and concise manner in which the webinars were presented. In regards to the presenters, having access to diverse experts and colleagues in public health who developed the methods/tools and used the method/tools, respectively, is also an important aspect of the webinars. While the overall content of the webinars is seen as relevant to respondents work, it is the *user stories* that have received overwhelming positive feedback. Participants repeatedly indicate that they appreciated that the user stories are “*real-life*” and “*practical*” examples that were very much applicable to their own work. Finally, the opportunity to ask questions of the experts and their colleagues was also identified as a strength of the webinars.

## Discussion

As webinars have been identified as an effective strategy to achieve reach in the dissemination of information (13, 15), NCCMT has used webinars to promote a rigorously identified collection methods and tools to support public health professionals consider best available research, along with other types of knowledge, in decision-making (2, 10). Evaluating our use of webinars within this context has provided our team with evidence that webinars increase awareness and stimulate the use of the collection of methods and tools within the Registry.

From this evaluation, we have gained insight into the valuable aspects of using webinars to disseminate information. Despite technological challenges (e.g., difficulty with the audio, being blocked from the webinar platform) (18–20, 24, 25, 27), webinars provide us the opportunity to reach a large number of public health professionals working in various roles across Canada and in some instances worldwide (14–20). Also, our webinars encourage participation, through the use of *polling questions*, *chat*, and *Q&A* functions of the webinar platform. This contributes to interaction and overwhelming positive experiences of attendees (14, 16, 19, 24, 25).

Both our experiences and the literature support webinars as an avenue for professional development (14). For example, recently, webinars have been used to deliver continuing education for pharmacists (24), inform health-care providers and the public about Ebola (19), provide training on HIV to health-care professionals, ministries, and non-government organizations (18), share information about pain research to researchers (17), support application of evidence among practitioners, researchers, students, managers, and policy makers in the field of mental health rehabilitation (27), and stimulate uptake of new Canadian guidelines about physical activity and sedentary behavior by organizations (26). Similar to Rhoads and colleagues (19), our findings indicate that the webinars are useful, relevant, and provide practical information in a way that is well organized, informative, and easy to understand. Also, in our follow-up survey, we found that a high percentage of respondents (42.1%) attended a previous webinar in our Spotlight webinar series, similar to findings of Buxton and colleagues (24). Another important aspect of our webinars is that attendees consistently viewed the webinars as an opportunity to learn from the experts who develop the methods/tools, which is consistent with Reid and colleagues (18), who found that the key reason people attended their webinars was to experience expert teaching from leading HIV researchers. Most importantly, webinars stimulate awareness and action about information. Similar to Gainforth and colleagues (26), who found that organizations attending webinars were more likely to post guidelines on their website than those not attending, our webinars increase awareness and intention to use information and stimulate action about the information presented in the webinars.

Given the valuable aspects webinars provide, it is our recommendation that webinars can continue to be used for professional development in general and specifically related to EIDM and knowledge translation (21). This is especially important given the identification of EIDM and research use in core competencies and standards for public health practice in Canada and worldwide (28–34). In addition to providing evidence for continuing the Spotlight webinars series, this evaluation informs how NCCMT can use webinars moving forward to disseminate information about our other resources to support EIDM and knowledge translation. For example, there is potential cost-savings in conducting webinars (13, 14, 20). The NCCMT resource costs associated with webinars is ~8 h of staff time ($400/webinar) plus $399/month for the webinar platform to reach on average 90 attendees are present per webinar. Whereas the estimated costs associated with a half-day in-person workshop for 20 attendees facilitated by one senior team member ranges from $1000 to $2000 per workshop depending on geographical location; costs includes printing, travel, and staff time for preparation (~4 h) and facilitation (~4 h).

### Lessons Learned

To assist individuals and/or organizations considering the use of webinars, there are important lessons learned from our 4 years implementing of our Spotlight webinar series. First is broadcasting the audio via the webinar platform rather than having participants join by teleconference; this has addressed attendee concerns that 90 min is too long to join by phone and eliminates distracting drawbacks such as echo, static, feedback, and/or noise during the webinar. In our experiences, broadcasting the audio has not hindered attendee interaction; when having the option to join *via* teleconference attendees have chosen to continue to post comments and questions in the *chat* and *Q&A* instead of posing them by phone. In addition to considering a webinar platform that allows for broadcasting of audio, we have found that a webinar platform which allows for the use and ability to accurately collect quantitative and qualitative data associated with the polling questions, chat, and Q&A functions is important. These interactive components are a helpful substitute for assessing engagement, awareness, and usefulness of the content in the absence of face-to-face contact. Being able to use these functions within the webinar is helpful for the presenter(s) to assess the audience both in real-time and following the webinar, which is key for the data analysis informing our evaluation of the webinars. This is particularly important given the low response rate to our follow-up survey (14.7–26.7%). Also, with attendees increasingly joining in groups (18), using polling questions to identify whether attendees are joining as individuals or groups allows us to more accurately identify webinar attendance. Using a platform that allows for advanced registration is also helpful. Not only does it allows us to communicate with registrants prior to and following the webinar (including disseminating the follow-up evaluation survey) but we are able to use registration rates as a measure of gauging overall interest in the webinar topic.

In addition, in our experience, not being able to access the webinar platform is commonly a firewall issue stemming from the location from where people are joining us, which is typically their workplace. Despite using different webinar platforms during our implementation of the Spotlight webinar series, people continue to experience this issue. Although not optimal, to address this technological challenge, we archive the PowerPoint and audio recording so that people can view and hear the webinar, asynchronously.

With different webinar platform options available, including those that are freely accessible (13, 14), choosing a platform is a crucial decision. Although our current platform (WebEx™) costs $399/month, we use it because it provides for the functions that we have identified as necessary and important to implementing our webinars. These include advance registration, accommodation of up to 250 attendees at one time, audio broadcasting (which has reduced our teleconference costs), polling questions/chat/Q&A, and data collection.

Finally, having a structure that identifies roles and responsibilities for implementation has also been a lesson learned. Having a communications coordinator who is responsible for all promotion of the webinar allows for consistent messaging within and across webinars. As it can be challenging to host a webinar while at the same time monitoring and responding to questions and comments posted by attendees, we have found involving a research assistant who is familiar with the webinar platform and is responsible for the technological aspects of the webinar, posting information in the chat, and monitoring the Q&A on the *day-of* the webinar is key. A senior level coordinator for the webinar series is also important. In addition to overseeing and coordinating all activities related to the webinar (from promotion to data analysis), their seniority allows for effective contact with the presenters and hosting of the webinars. In conducting webinars in which audio is broadcasted and attendees only post comments/questions to the webinar platform, our host plays an important role in also monitoring the chat and Q&A to read aloud attendee comments and questions for response by the presenters.

### Limitations

Although this evaluation provides insights into the value of using webinars, some limitations should be noted. First, because the follow-up survey is sent to all webinar registrants, it is possible that the responses to this survey do not reflect only webinar attendees. In addition, despite the use of a reminder e-mail at 2 weeks, there was a low response rate to the 6-month survey, with only 46 (15.1%) webinar attendees of the two webinars responding. As such, the findings from the 6-month survey should be interpreted with a degree of caution. Finally, Google Analytics data used to determine the page views of the methods/tools before and after the webinar are not based only on the traffic of webinar registrants/attendees; the data reflect all traffic to the web pages.

## Conclusion

The National Collaborating Centre for Methods and Tools Spotlight on Knowledge Translation Methods and Tools webinar series increases awareness of and stimulates action about resources that can be used to support EIDM and knowledge translation in public health decision-making. These webinars increase the reach of expert, high quality information about such resources to large and varied audiences. Evidence suggests that webinars will continue to be a useful strategy for the transfer of knowledge about EIDM among public health and other health-care professionals to in turn implement effective health services, to achieve positive health outcomes (10).

## Author Contributions

MD currently is the Scientific Director for NCCMT; thus, responsible for overseeing all work related to the Registry of Methods and Tools. MD, JM, KR, and JY are responsible for the implementation of the webinars. MD assists in prioritizing identified methods/tools for webinars. JM (1) assists in identification of methods/tools for webinars (including generation of Google Analytics and collection of *user stories*) and is responsible for (2) promotion of the webinars, which includes reviewing the one-pager, writing of promotional material included in the newsletter and Tweets, and disseminating the newsletter and Tweets, (3) dissemination of PowerPoint and audio files following the webinar, and (4) collection of Google Analytics data. KR is responsible for the technological aspects of the webinar platform. JY is responsible for (1) identifying webinar topics, (2) all contacts with webinar presenters, (3) drafting the one-pager, (3) moderating webinars, (4) follow-up survey data collection, and (5) the additional evaluation study. JY drafted the initial manuscript. All authors contributed to revision and final approval of the manuscript.

## Conflict of Interest Statement

The authors declare that this project was conducted in the absence of any commercial or financial relationships that could be construed as a potential conflict of interest.

## Footnotes

^1^http://nccmt.ca/resources/about

^2^http://nccmt.ca/professional-development/event-calendar#events_calendar

^3^http://nccmt.ca/resources/publications?entity=pub&q=&locale=en&filters=242
